# Small Bowel Obstruction Caused by Meckel’s Diverticulum Entrapped behind Transperitoneal Sigmoid Colostomy: A Case Report

**DOI:** 10.70352/scrj.cr.25-0794

**Published:** 2026-03-05

**Authors:** Hidenobu Nakagama, Koji Tamura, Takaaki Fujimoto, Kinuko Nagayoshi, Yusuke Mizuuchi, Kenoki Ohuchida, Masafumi Nakamura

**Affiliations:** Department of Surgery and Oncology, Kyushu University, Fukuoka, Fukuoka, Japan

**Keywords:** Meckel’s diverticulum, transperitoneal colostomy, small bowel obstruction, minimally invasive surgery, laparoscopic surgery

## Abstract

**INTRODUCTION:**

Meckel’s diverticulum is the most common congenital anomaly of the gastrointestinal tract, often asymptomatic but occasionally causing inflammation, bleeding, or intestinal obstruction. Permanent colostomy after abdominoperineal resection (APR) is typically created via either a transperitoneal or extraperitoneal route. Although small bowel obstruction (SBO) is a known complication of transperitoneal colostomy, SBO caused by Meckel’s diverticulum entrapped behind the stoma has not previously been reported. We describe an extremely rare case of SBO caused by adhesion between Meckel’s diverticulum and the sigmoid colon at the stoma site.

**CASE PRESENTATION:**

A 70-year-old man underwent robot-assisted APR with transperitoneal sigmoid colostomy for rectal cancer (pT3N0M0, UICC 9th edition). Ten months later, he presented with progressive abdominal pain and distension. CT demonstrated a caliber change without evidence of strangulation, suggesting adhesive SBO. Conservative management with nasogastric decompression and a long intestinal tube failed, and aspiration pneumonia developed during tube placement, temporarily delaying surgery. After recovery, laparoscopic exploration was performed. A 6-cm Meckel’s diverticulum located approximately 1 m proximal to the ileocecal valve was found to be firmly adherent to the elevated sigmoid colon at the stoma site. The dilated small bowel loops were entrapped behind the colostomy segment, creating an obvious transition point. Laparoscopic adhesiolysis and diverticulectomy were successfully completed. The patient recovered uneventfully, resumed oral intake on POD 6, and has had no recurrence of SBO. Histopathology confirmed Meckel’s diverticulum.

**CONCLUSIONS:**

This case demonstrates a rare mechanism of SBO caused by Meckel’s diverticulum trapped behind a transperitoneal colostomy. From the anatomical perspective, the extraperitoneal route could reduce the risk of adhesion-related complications compared to the transperitoneal route. Laparoscopy served as an effective dual-purpose modality for both diagnosis and minimally invasive treatment when the etiology of SBO is uncertain.

## Abbreviations

APR
: abdominoperineal resection
CRP
: C-reactive protein
SBO
: small bowel obstruction

## INTRODUCTION

Meckel’s diverticulum, a vestigial remnant of the vitelline duct, is the most common congenital anomaly of the gastrointestinal tract, occurring in approximately 0.3%–4.0% of the population.^1)^ It is sometimes identified incidentally without any symptoms during surgery for other abdominal diseases. On the other hand, some patients with Meckel’s diverticulum present with a range of clinical symptoms, making preoperative diagnosis difficult.^2)^

A permanent colostomy is typically performed during APR for low rectal cancer, and 2 principal approaches are used for colostomy creation: transperitoneal and extraperitoneal colostomy. Although transperitoneal colostomy is widely adopted because of its technical simplicity and reliable blood supply, it carries a risk of stoma-related complications.^3)^ SBO is a common complication associated with transperitoneal colostomy; however, to our knowledge, there have been no reports of SBO caused by Meckel’s diverticulum entrapped behind the stoma site colon.

We herein present a rare case of SBO caused by an unusual adhesion mechanism between Meckel’s diverticulum and the sigmoid colon at the stoma site.

## CASE PRESENTATION

**Fig. 1:**
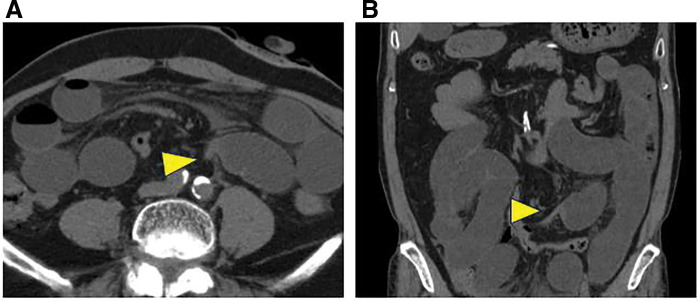
Preoperative CT imaging. Preoperative CT revealed a caliber change in bowel dilatation in the left middle abdomen (**A**, axial section; **B**, coronal). No significant signs of strangulation were identified, and the postoperative diagnosis was mechanical small-bowel obstruction. The arrowheads indicate the caliber change at the transition point of obstruction.

A 70-year-old man was diagnosed with rectal cancer and underwent robot-assisted APR with transperitoneal colostomy. The patient was receiving continuous aspirin therapy for significant cardiovascular comorbidities, including a history of myocardial infarction, carotid artery stenosis, and peripheral arterial occlusive disease; the tissues were fragile and prone to bleeding during surgery. Therefore, a transperitoneal route was selected, as simple transperitoneal elevation of the colon was considered to reduce the risk of bleeding and tissue injury and to shorten the operative time compared with an extraperitoneal route. At that time, the presence of Meckel’s diverticulum could not be recognized intraoperatively. The postoperative pathological diagnosis was pT3N0M0, pStage IIA (UICC 9th edition). He underwent postoperative surveillance without adjuvant therapy, and no recurrence was observed. Ten months later, he presented to the emergency department with abdominal pain. Physical examination revealed abdominal distension with spontaneous pain and tenderness in the upper abdomen. Laboratory tests showed a white blood cell count of 7900/μL and a CRP level of 0.47 mg/dL, without elevation in inflammatory markers. CT images revealed a caliber change without apparent evidence of strangulation, suggesting adhesive SBO (**Fig. 1A** and **1B**). Furthermore, CT images did not reveal the presence of any diverticulum.

The patient was admitted and initially managed with nasogastric decompression. Because neither the symptoms nor the abdominal gas patterns on X-ray improved, a long intestinal decompression tube was inserted on the following day. During tube placement, the patient aspirated, developing aspiration pneumonia, which required antibiotic therapy and respiratory support for 1 week. Despite conservative therapy with the long intestinal decompression tube, his symptoms persisted. After improvement of the pneumonia, surgical treatment was planned without performing additional examinations, such as contrast imaging, and laparoscope-assisted adhesiolysis was performed.

**Fig. 2:**
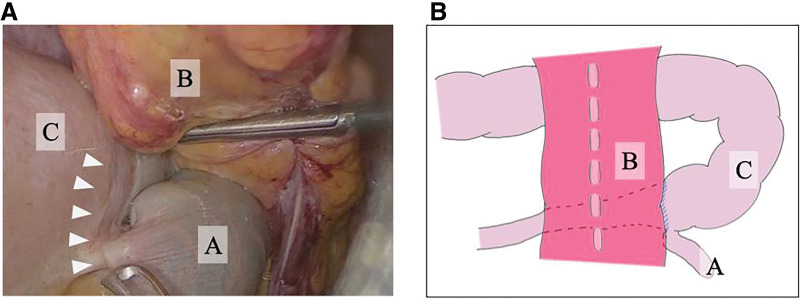
Intra-abdominal findings on laparoscopic view. (**A**) Meckel’s diverticulum (A) was found entrapped behind the transperitoneal colostomy (B). This entrapment created a focal narrowing of the small intestine (C), serving as the transition point leading to small-bowel obstruction (white arrowheads). (**B**) Schematic illustration of the laparoscopic findings.

**Fig. 3:**
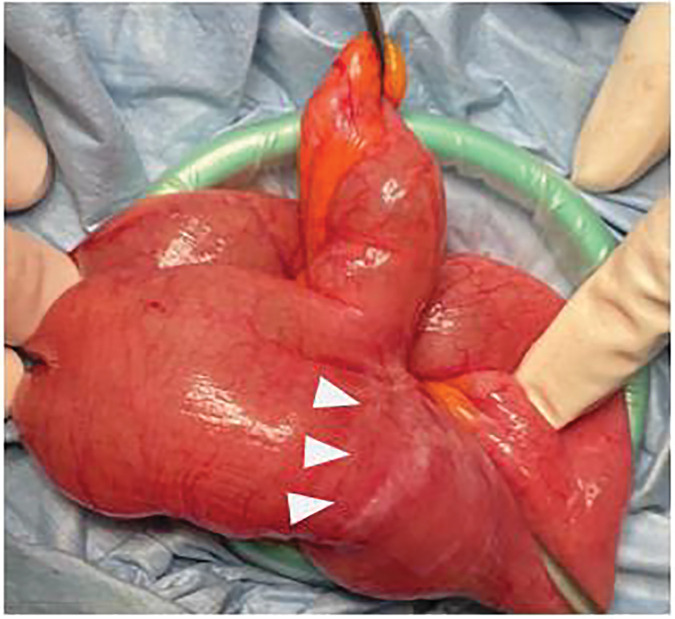
Extracorporeal operative findings showing resection of Meckel’s diverticulum. No other apparent cause of small-bowel obstruction was identified. The Meckel’s diverticulum was resected. White arrowheads indicate the transition point responsible for the obstruction.

Intraoperatively, a Meckel’s diverticulum approximately 6 cm in length, which had not been recognized in the previous surgery, was identified 1 m proximal to the ileocecal valve. It was circumferentially adherent to the elevated sigmoid colostomy segment, entrapping the dilated small bowel behind the stoma-site colon and creating an obvious transition point (**Fig. 2A** and **2B**). Adhesiolysis and diverticulectomy were successfully performed (**Fig. 3**). The postoperative course was uneventful: the long tube was removed on POD 3, and oral intake was resumed on POD 6. Histopathological examination confirmed Meckel’s diverticulum. No recurrence of bowel obstruction has been observed to date.

## DISCUSSION

In the present case, Meckel’s diverticulum was entrapped and adhered behind the transperitoneal sigmoid colostomy, resulting in SBO. This mechanism is extremely rare, and to the best of our knowledge, this is the first case report of SBO caused by entrapment of Meckel’s diverticulum behind a stoma created via the transperitoneal route.

Although Meckel’s diverticulum is present in a small proportion of the population and is often asymptomatic, complications such as inflammation, bleeding, and intestinal obstruction may occur.^1)^ Bowel obstruction is more frequently observed in children,^4)^ and although rare in adults, is most commonly caused by adhesions.^5)^ In the present patient, the postoperative anatomical arrangement following transperitoneal colostomy likely facilitated adhesion between the colostomy segment and Meckel’s diverticulum, leading to obstruction. This rare mechanism highlights the importance of recognizing potential anatomical anomalies and adhesion-prone configurations during stoma construction.

Two principal approaches are used for single-barrel colostomy creation: transperitoneal and extraperitoneal. Although no statistically significant difference in the overall incidence of SBO has been established between the 2 techniques, extraperitoneal colostomy has been reported to be associated with fewer stoma-related complications—including parastomal hernia, stomal prolapse, mucocutaneous separation, and stoma retraction—compared with transperitoneal colostomy.^6–8)^ Although high-level evidence remains lacking, extraperitoneal colostomy may reduce the risk of SBO.^7)^

In transperitoneal colostomy, a single-lumen stoma inevitably creates a lateral intraperitoneal space on the side adjacent to the elevated bowel segment. This free space allows small bowel loops to migrate and become entrapped, thereby predisposing patients to adhesion formation and subsequent bowel obstruction. In contrast, extraperitoneal colostomy routes the bowel through the extraperitoneal space, minimizing its exposure to intra-abdominal contents such as Meckel’s diverticulum and potentially reducing the risk of adhesion-related SBO. Furthermore, the extraperitoneal route offers the advantage of greater fixation of the bowel, which may minimize the risks of torsion and luminal narrowing. Although the extraperitoneal route has potential advantages, it is associated with several limitations, including a higher incidence of defecation sensation and greater technical complexity, particularly in laparoscopic surgery. In addition, the procedure may show limited reproducibility depending on surgeon’s experience and institutional expertise. Therefore, patient anatomy and the surgeon’s technical proficiency should be carefully considered when selecting the optimal stoma route.

Recently, the number of target organs and indications for laparoscopic surgery has gradually expanded, and reduced rates of both intraoperative and postoperative complications have been reported.^9)^ Furukawa et al. reported that laparoscopic surgery for SBO provides better short-term and comparable long-term outcomes compared with open surgery.^10)^ In the present case, although the patient had a history of prior abdominal surgery, the initial operation had also been performed using a minimally invasive surgery, and sufficient bowel decompression was achieved with a long intestinal tube. These factors supported our decision to adopt a laparoscopic approach. As a result, we were able to inspect the entire abdominal cavity, promptly identify Meckel’s diverticulum as the unusual cause of SBO, and successfully perform adhesiolysis and diverticulectomy. Diagnostic laparoscopy offers distinct advantages as an initial surgical strategy for SBO of uncertain etiology, as it serves both diagnostic and therapeutic purposes with minimal invasiveness.

## CONCLUSIONS

This case highlights a rare mechanism of SBO caused by an entrapped Meckel’s diverticulum behind a transperitoneal colostomy. It underscores the potential role of extraperitoneal colostomy in minimizing adhesion-related complications. A laparoscopic approach should be considered a valuable dual-purpose modality in the management of SBO, serving both as a diagnostic tool to elucidate the underlying cause and as a minimally invasive therapeutic technique.

## DECLARATIONS

### Availability of data and materials

All data in this article are available within the article.

## References

[1] Yahchouchy EK, Marano AF, Etienne JC, et al. Meckel’s diverticulum. J Am Coll Surg 2001; 192: 658–62. 11333103 10.1016/s1072-7515(01)00817-1

[2] Yamaguchi M, Takeuchi S, Awazu S. Meckel’s diverticulum: investigation of 600 patients in Japanese literature. Am J Surg 1978; 136: 247–9. 308325 10.1016/0002-9610(78)90238-6

[3] Portales Rivera CM. Effectiveness of extraperitoneal colostomy for the prevention of complications compared to the transperitoneal technique: literature review and statistical analysis (Preprint). Authorea 2025.

[4] Leijonmarck CE, Bonman-Sandelin K, Frisell J, et al. Meckel’s diverticulum in the adult. Br J Surg 1986; 73: 146–9. 3484984 10.1002/bjs.1800730225

[5] Choi S-Y, Hong SS, Park HJ, et al. The many faces of Meckel’s diverticulum and its complications. J Med Imaging Radiat Oncol 2017; 61: 225–31. 27492813 10.1111/1754-9485.12505

[6] Luo J, Singh D, Zhang F, et al. Comparison of the extraperitoneal and transperitoneal routes for permanent colostomy: a meta-analysis with RCTs and systematic review. World J Surg Oncol 2022; 20: 82. 35279174 10.1186/s12957-022-02547-9 PMC8918274

[7] Lian L, Wu X-R, He X-S, et al. Extraperitoneal vs. intraperitoneal route for permanent colostomy: a meta-analysis of 1,071 patients. Int J Colorectal Dis 2012; 27: 59–64. 21892608 10.1007/s00384-011-1293-6

[8] Isah AD, Wang X, Shaibu Z, et al. Systematic review and meta-analysis comparing extraperitoneal and transperitoneal routes of colostomy-related complications. World J Gastrointest Surg 2025; 17: 98947. 40162385 10.4240/wjgs.v17.i3.98947 PMC11948114

[9] Shiroshita H, Inomata I, Takiguchi S, et al. Update on endoscopic surgery in Japan: result of the 16th National Survey of endoscopic surgery by the Japan Society for Endoscopic Surgery. Asian J Endosc Surg 2024; 17: e13285. 39235764 10.1111/ases.13285

[10] Furukawa S, Kato K, Susa Y, et al. Is exploratory laparoscopy the optimal surgical strategy for small bowel obstruction? A single-center retrospective cohort study with propensity score-matched analysis. Asian J Endosc Surg 2025; 18: e70056. 40189409 10.1111/ases.70056

